# The Difference of Milk-Derived Extracellular Vesicles from Cow Colostrum and Mature Milk on miRNAs Expression and Protecting Intestinal Epithelial Cells against Lipopolysaccharide Damage

**DOI:** 10.3390/ijms25073880

**Published:** 2024-03-30

**Authors:** Wenju Liu, Chao Du, Liangkang Nan, Chunfang Li, Haitong Wang, Yikai Fan, Shujun Zhang

**Affiliations:** 1 Key Lab of Agricultural Animal Genetics, Breeding and Reproduction of Ministry of Education, Huazhong Agricultural University, Wuhan 430070, China; liuwenju2020@webmail.hzau.edu.cn (W.L.); dc1992hml@163.com (C.D.); 15827557518@163.com (L.N.); chunfangli0521@126.com (C.L.); wang00411@163.com (H.W.); fanyikai123@126.com (Y.F.); 2 Frontiers Science Center for Animal Breeding and Sustainable Production of Ministry of Education, Huazhong Agricultural University, Wuhan 430070, China

**Keywords:** dairy cow, EVs, colostrum and mature milk, miRNAs, apoptosis, proliferation, intestinal epithelial barrier

## Abstract

Intestinal epithelial cells (IECs) play crucial roles in forming an essential barrier, providing host defense against pathogens and regulating nutrients absorption. Milk-derived extracellular vesicles (EVs) within its miRNAs are capable of modulating the recipient cell function. However, the differences between colostrum and mature milk EVs and their biological function in attenuating intestinal epithelial cell injury remain poorly understood. Thus, we carried out the present study to characterize the difference between colostrum and mature milk-derived miRNA of EVs and the effect of colostrum and mature milk EVs on the proliferation, apoptosis, proinflammatory cytokines and intestinal epithelial barrier related genes in IEC-6 induced by LPS. Differential expression of 329 miRNAs was identified between colostrum and mature milk EVs, with 185 miRNAs being downregulated and 144 upregulated. In addition, colostrum contains a greater number and protein concentration of EVs than mature milk. Furthermore, compared to control, EVs derived from colostrum significantly inhibited the expression of apoptosis- (*Bax*, *p53*, and *caspase-3*) and proinflammatory-related genes (*TNFα*, *IL6*, and *IL1β*). EVs derived from mature milk did not affect expression of apoptosis-related genes (*Bax*, *p53*, *bcl2*, and *caspase-3*). The EVs derived from mature milk significantly inhibited the expression of proinflammatory-related genes (*TNFα* and *IL6*). Western blot analysis also indicated that colostrum and mature milk EVs significantly decreased the apoptosis of IEC-6 cells. The EdU assay results showed that colostrum and mature milk EVs significantly increased the proliferation of IEC-6 cells. The expression of intestinal barrier-related genes (*TJP1*, *CLDN1*, *OCLN*, *CDX2*, *MUC2,* and *IGF1R*) was significantly promoted in IEC-6 cells after colostrum and mature milk EVs addition. Importantly, colostrum and mature milk EVs significantly relieved the LPS-induced inhibition of proliferation and intestinal barrier-related genes expression and attenuated apoptosis and proinflammatory responses induced by LPS in IEC-6 cells. Flow cytometry and Western blot analysis also indicated that colostrum and mature milk EVs significantly affect the apoptosis of IEC-6 cells induced by LPS. The results also indicated that EVs derived from colostrum had better effects on inhibiting the apoptosis- and proinflammatory cytokines-related genes expression. However, the EVs derived from mature milk exhibited beneficial effects on intestinal epithelial barrier protection. The present study will provide a better understanding of the role of EVs derived from colostrum and milk in dairy cows with different responses in the regulation of intestinal cells function, and also presents new evidence for the change of EVs cargos during various stages of lactation.

## 1. Introduction

The gastrointestinal tract is not only responsible for the first physiological step of transporting nutrients to the body’s cells but also plays an essential role in the regulation of the development and health of infants [1,2]. Nutrition plays a critical role in the functional growth and maturation of the gastrointestinal tract [3]. Intestinal epithelial cells form an essential barrier and affect the absorption of nutrients [4]. However, due to the greatest exposure to the outside environment, intestinal epithelial cells usually undergo infection with pathogens, such as intact bacteria and bacterial products, which disrupt the intestinal mucosa integrity and pass through the barrier, enter the blood, cause the translocation of bacteria and endotoxins, promote the occurrence of enteric infections, and even develop systemic inflammatory response syndrome [5,6]. Importantly, intestinal epithelial cells, the largest component of the innate immune system, can respond quickly to intestinal microbial invasion [7]. Therefore, the intestinal epithelium plays a critical role in the inflammatory response and host defense against pathogens, which can ensure gut barrier integrity [8].

Apart from providing an optimal source of nutrition, milk also contains many bioactive components, primarily consisting of microbes, growth factors, oligosaccharides, hormones, immunoglobulins, lactoferrin and fatty acids, which play a critical role in the establishment of the intestinal microbiome and ultimately influence intestinal inflammation and gut health [9,10,11]. Recently, there has been increasing evidence concerning milk EVs’ role in regulating gut health. The EVs with membranous vesicles are secreted by multiple cell types [12,13]. A major characteristic of EVs is that it is involved in the intercellular communication by transferring its proteins, lipids, and RNAs to the recipient cell and then mediating the cell’s response [12,13,14]. In addition, many milk miRNAs are packaged into EVs, and the membrane-shaped structure of EVs is critical to miRNAs’ stability and resistance to harsh conditions, such as 37 °C for >1 h, acidic pH, and the presence of RNase [15,16,17,18]. Therefore, EVs ensure the miRNAs can pass through the gastrointestinal tract, be absorbed in the intestine and reach the systemic circulation, where they exert functional effects [19,20,21,22]. In recent years, EVs have been successfully isolated and identified from milk [23,24], and milk-derived EVs have attracted interest because of their advantages of being readily available, stable under long-term storage condition, generally safe and a good source [25]. Moreover, it is believed that EVs miRNAs are responsible for the beneficial effects of EVs in promoting immune function, reducing inflammation and attenuating intestinal epithelial cell injury [26,27].

It is well known that miRNAs are involved in regulating gene expression by binding to the target mRNAs, which results in a reduction in the expression of target mRNAs. Numerous miRNAs, including the immune-related miRNAs, have been found in colostrum and milk [15,28,29], and enrichment evidence has showed that dairy cow colostrum and milk miRNA are particularly packed into colostrum- and milk-derived EVs [15,17,18,29,30]. Milk EVs also play an essential role in promoting intestinal tolerance. It has been proved that milk EVs miRNA-148a-3p targets *DNMT1* mRNA and miRNA-148a-3p are conserved between Bos taurus and Homo sapiens milk-derived EVs [31,32,33]. Researchers have shown that miRNA-148a-3p enhances FOXP3 demethylation, which enhances the expression of FoxP3+ regulatory T cell (Treg) by targeting *DNMT1*. The Treg-specific-demethylation region (TSDR) on the promoter of FOXP3 is an important methylation-sensitive element regulating Foxp3 expression and demonstrates that epigenetic imprinting in this region is critical for establishment of a stable Treg lineage [34,35]. Thus, milk EVs play an important role in *DNMT1* suppression and Treg development in the intestine [36]. Moreover, EVs and their miRNAs are also reported to serve as a novel functional component of milk to promote intestinal epithelial cell viability and improve intestinal epithelial barrier function after injury in yaks [16], bovines [17,37], pigs [5], and camels [38]. Therefore, the EVs within milk’s miRNAs are capable of modifying the specific genes expression and then modulating the recipient cell function. 

However, the biological function of cow EVs derived from colostrum and mature milk on regulating epithelial barrier and inflammation caused by LPS (lipopolysaccharide), and the difference between colostrum and mature milk EVs on attenuating intestinal epithelial cell injury remain poorly understood. Thus, we carried out the present study to simulate intestinal injury using IEC-6 cells under LPS treatment, and to investigate the effect of the EVs derived from colostrum and mature milk with these injured IEC-6 cells. In addition, we aimed to compare the difference in EVs derived from bovine colostrum and mature milk on mediating the IEC-6 cells’ function, the miRNA expression, the number and protein concentration of EVs. Therefore, the present study will provide a better understanding the role of EVs derived from colostrum and mature milk in dairy cows with different responses to regulation of intestinal tract development, and also present new evidence for the change of EVs cargos during various stages of lactation.

## 2. Results

### 2.1. Characterization of EVs Derived from Colostrum and Mature Milk

**Figure 1:**
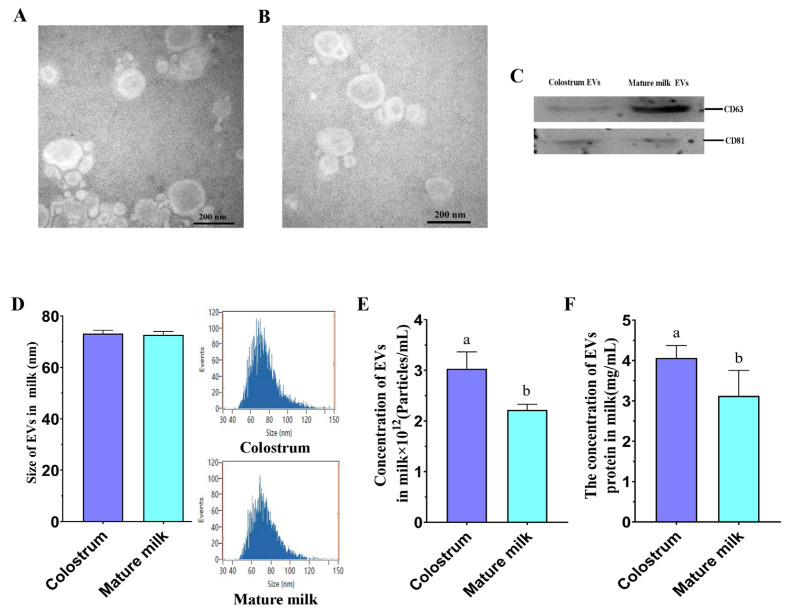
Characterization of the isolated EVs from cow colostrum and mature milk after ultracentrifugation. (**A**,**B**) Transmission electron microscopic (TEM) image of EVs in colostrum (**A**) and mature milk (**B**). (**C**) Western blot of known EVs markers (CD63 and CD81). (**D**) Nano-flow cytometry analysis showing particle size distribution in EVs derived from cow colostrum and mature milk. (**E**) Nano-flow cytometry showing the concentration of EVs isolated from cow colostrum and mature milk. (**F**) The protein concentration of EVs isolated from cow colostrum and mature milk. Bars with different lowercase letters indicate significant difference (*p* < 0.05). Data are presented as the mean ± standard deviations (SD).

The EVs derived from cow’s milk were identified by TEM and Western blot. The TEM showed the EVs morphology with the cup-shaped lipid layers structure (Figure 1A,B, Figure S1). In addition, the EVs protein markers (CD81 and CD63) also were detected by Western blot (Figure 1C, Figure S2), suggesting that cow milk EVs were successfully isolated with ultracentrifugation. The colostrum and mature milk-derived EVs were approximately 73 nm in diameter (Figure 1D). Then, the characteristics of the EVs derived from colostrum and mature milk were investigated by detecting the number and protein concentration of EVs. The number of EVs and EVs protein concentration were significantly higher in colostrum compared with those in mature milk (Figure 1E,F). This suggested that colostrum contain more EVs than mature milk.

### 2.2. Identification of Differentially Expressed miRNA

RNA-seq was performed to obtain new and published miRNAs expressed in cow colostrum and mature milk EVs. A total of 97,717,760 clean reads for cow colostrum EVs and 90,740,728 clean reads for cow mature milk EVs were detected, respectively. The miRNAs length distribution mainly ranged from 16 to 30 nt, with 22 nt accounted for the highest number. Based on their biogenesis and annotations, the detected miRNAs were categorized into different kinds of small RNA species (Supplementary Table S2). We also found that the top 40 miRNAs in cow colostrum and mature milk EVs participated in regulating intestinal barrier function and inflammation by involving in related signaling pathways (Figure 1A,B). Moreover, the further analysis revealed that 36 miRNAs, ranked among the top 40 most abundant miRNAs associated with intestinal barrier integrity were present in both cow colostrum and mature milk EVs (Figure 1B,C). The presence of those abundant miRNAs suggests that colostrum and mature milk EVs may exert similar functions in regulating intestinal barrier integrity.

**Figure 2:**
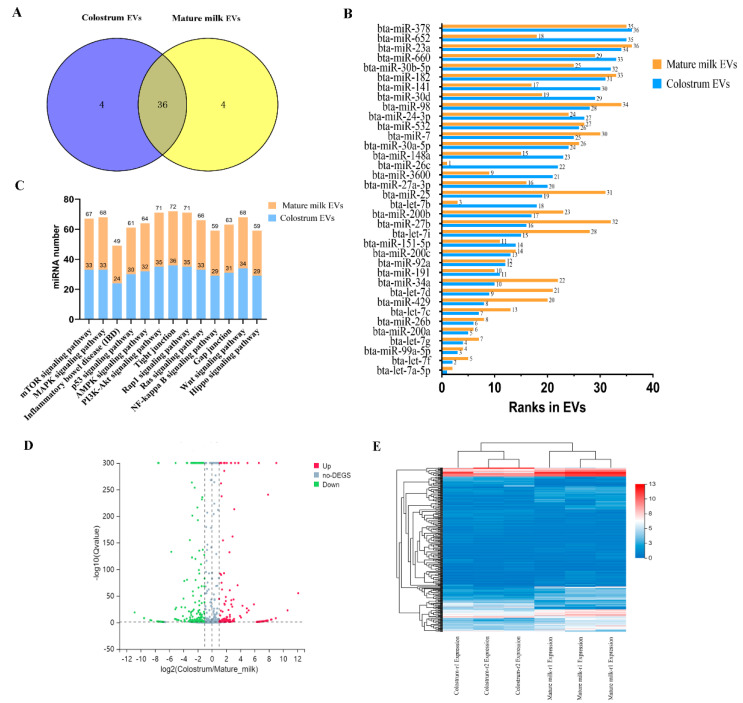
Identification of differential miRNAs in cow colostrum and mature milk EVs. (**A**) Venn diagrams of top 40 microRNAs in cow colostrum and mature milk EVs. (**B**) Ranks in the top 36 miRNAs in colostrum and mature milk EVs. (**C**) *Kyoto Encyclopedia of Genes and Genomes* (KEGG) pathway analysis of cow colostrum and mature milk EVs miRNAs associated with intestinal barrier function. (**D**) Volcano plots of milk EVs differentially expressed miRNAs between cow colostrum and mature milk. X-axis denotes fold change (log2); Y-axis refers to the q value (−log10). (**E**) Hierarchical clustering of differential miRNAs.

**Figure 3:**
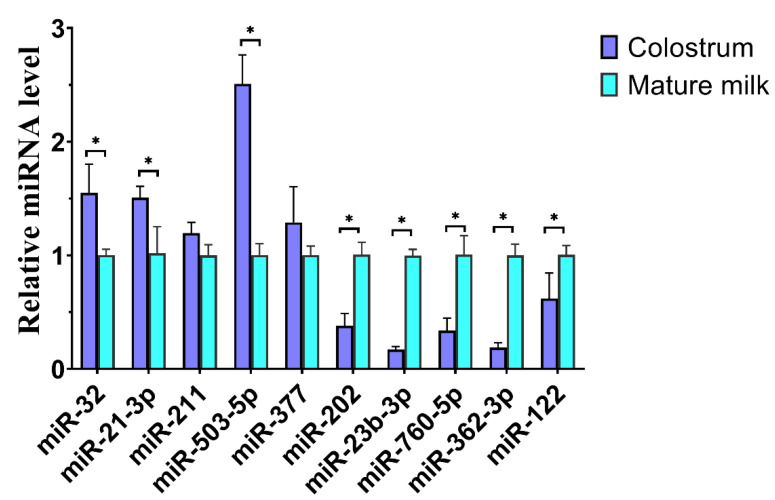
Quantitative verification results of miRNA. The relative expression of 10 miRNAs in cow colostrum and mature milk EVs samples was verified. The quantity of miRNA was normalized to that of U6. * represents significant difference (*p* < 0.05). The experiment was repeated three times independently.

To characterize miRNAs expression profiles, we detected miRNAs expression in EVs derived from colostrum and mature milk. After differential expression analysis, 329 miRNAs (210 known miRNAs and 119 novel miRNAs) were identified between colostrum and mature milk EVs (Supplementary Table S3). Among them, 185 miRNAs were significantly downregulated and 144 miRNAs were significantly upregulated in the EVs derived from colostrum compared with mature milk EVs (Figure 2D,E). To verify the accuracy of sequencing data of miRNAs in cow milk EVs, ten random selected differentially expressed miRNAs were detected by qPCR in colostrum and mature milk EVs (Figure 3). The qPCR result was consistent with the miRNA-sequencing data, showing that bta-miR-32, bta-miR-21-3p, and bta-miR-503-5p increased, while bta-miR-202, bta-miR-23b-3p, bta-miR-760-5p, bta-miR-362-3p, and bta-miR-122 decreased in colostrum compared to mature milk EVs, which verified the reliability of the miRNA-sequencing data in colostrum and mature milk EVs.

### 2.3. GO and KEGG Analysis of Differentially Expressed miRNA

**Figure 4:**
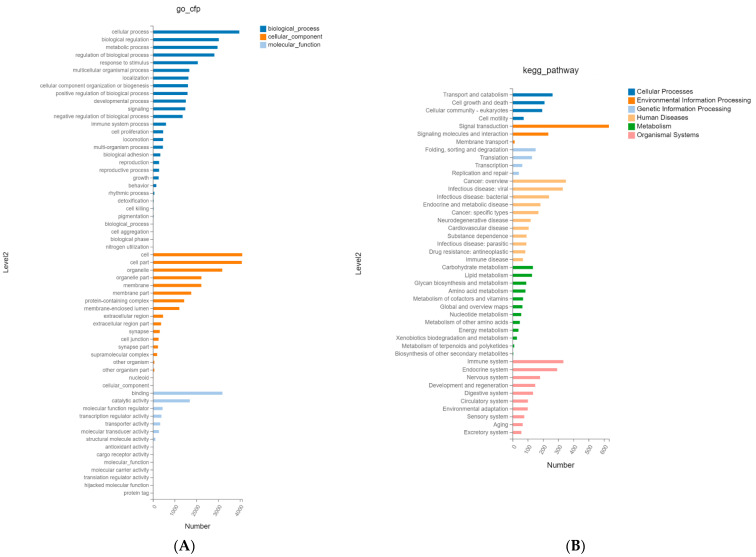
The gene ontology (GO) and KEGG enrichment analysis. (**A**) The GO enrichment analysis of differential expressed miRNAs. (**B**) The KEGG enrichment analysis of differential expressed miRNAs.

To further investigate the differentially expressed miRNAs involved in biological functions, the target genes significantly regulating miRNAs were predicted using the databases, including RNAhybrid, miRanda, and TargetScan. The GO enrichment analysis of the target genes showed that the target genes were mainly enriched for biological processes, such as cellular process, biological regulation, metabolic process, regulation of biological process, and response to stimulus (Figure 4A, Supplementary Table S4). Moreover, the target genes involved in the cellular components mainly included cell, cell part, organelle, membrane, protein-containing complex, and membrane-enclosed lumen. The target genes enriched in molecular functions terms were mainly associated with binding, catalytic activity, and molecular function regulation. 

**Figure 5:**
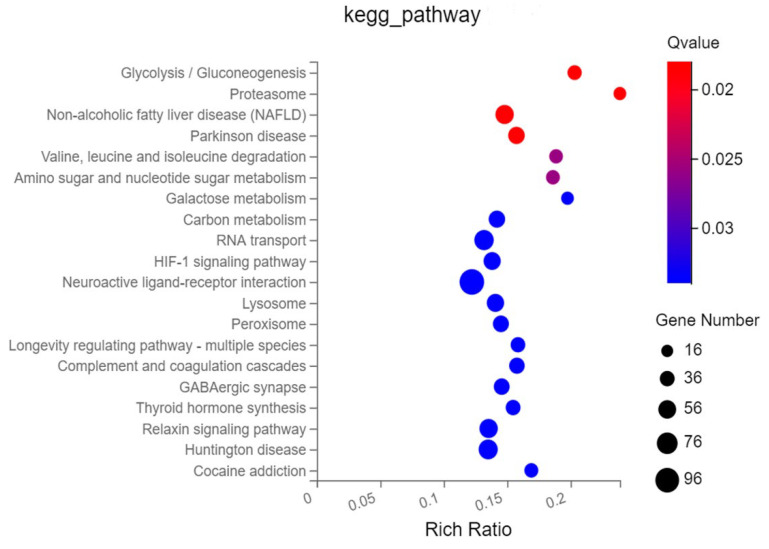
The top 20 KEGG enrichment analysis of differential expressed miRNAs.

KEGG functional analysis showed that they were mainly enriched in signal transduction, transport and catabolism, signaling molecules and interaction, cell growth and death, viral infectious disease, bacterial infectious disease, immune system, etc. (Figure 4B, Supplementary Table S5). The top 20 most-targeted pathways identified based on the KEGG pathway analysis in colostrum and mature milk EVs are shown in Figure 5. 

### 2.4. Cow Colostrum and Mature Milk EVs Attenuate LPS-Induced Proliferation Inhibition and Apoptosis in IEC-6 Cells

**Figure 6:**
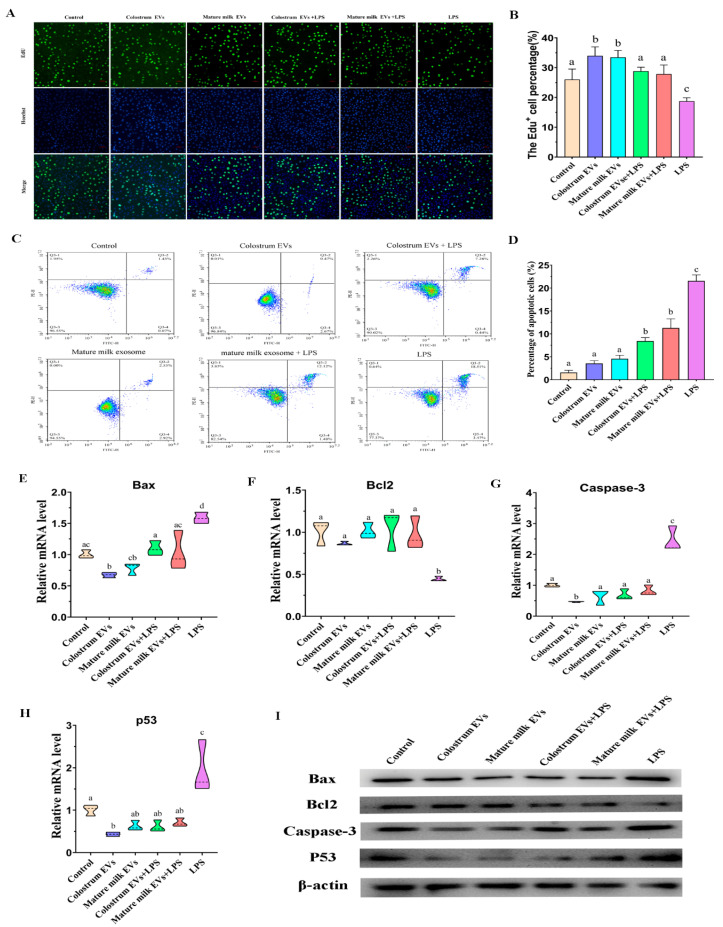
Effects of cow colostrum and mature milk EVs supplementation on proliferation and apoptosis in IEC-6 cells. (**A**) EdU staining of IEC-6 cells (n = 3), scale bar = 25 μm. (**B**) Statistical results of EdU staining data (n = 3). (**C**,**D**) The percentage of apoptotic IEC-6 cells as detected by flow cytometry. (**E**–**H**) Relative *Bcl2*, *Bax*, *p53,* and *Caspase-3* mRNA level in IEC-6 cells (n = 3); (**I**) Relative Bcl2, Bax, p53, and Caspase-3 protein level in IEC-6 cells (n = 3). The relative expression of mRNAs was normalized to β-actin levels. Significant differences were determined using one-way ANOVA. Results were presented as the mean ± standard deviations (SD). Bars with different lowercase letters indicate significant difference (*p* < 0.05).

To confirm whether cow colostrum and mature milk EVs could reduce LPS-induced injury in IEC-6 cells, the proliferation of IEC-6 cells was conducted by EdU fluorescence assay (Figure 6A,B). The results showed that cow colostrum and mature milk EVs both significantly increased the percentage of EdU-positive fluorescence cells compared with control (*p* < 0.05). Moreover, the percentage of EdU-positive fluorescence cells in the LPS group was lower than that in the control group, while cow colostrum and mature milk EVs significantly attenuated the inhibited effects caused by LPS (*p* < 0.05). There was no significant difference in the EdU-positive fluorescence cells between the cow colostrum EVs group and the mature milk EVs group (*p* > 0.05). Furthermore, the apoptosis of cells was quantified by flow cytometry (Figure 6C,D). Colostrum and mature milk EVs did not affect the IEC-6 cells apoptosis compared with control (*p* > 0.05). However, compared with the control group, the apoptosis of IEC-6 cells was significantly induced by LPS, while colostrum and mature milk EVs significantly reduced the apoptosis induced by LPS, which was still higher than the apoptosis of control. Furthermore, the expression of apoptosis-related genes was also detected by qPCR (Figure 6E–I, Supplementary Table S6, Figures S3–S7). Compared to the control, colostrum EVs significantly inhibited the expression of *Bax*, *p53*, and *Caspase-3* (*p* < 0.05), and mature milk EVs did not affect the expression of *Bax*, *p53*, and *Caspase-3* (*p* > 0.05), while LPS significantly increased the expression of *Bax*, *p53*, and *Caspase-3*, and inhibited the expression of *bcl2* (*p* < 0.05). With regard to the expression of *Bcl2*, colostrum and mature milk EVs did not change their expression compared with the control (*p* > 0.05). There was no significant difference in the apoptosis-related genes between the colostrum EVs group and the mature milk EVs group, except for *Caspase-3* (*p* < 0.05). However, western blot showed that colostrum and mature milk EVs could inhibited the expression of Bax, p53, and Caspase-3. Importantly, colostrum and mature milk EVs treatment reversed the effect of LPS on the expression of *Bax*, *p53*, *Bcl2,* and *Caspase-3* (*p* < 0.05). Flow cytometry and Western blot analysis also indicated that colostrum and mature milk EVs significantly affect the apoptosis of IEC-6 cells induced by LPS. These results suggested that colostrum and mature milk EVs could promote the proliferation and inhibit the apoptosis and attenuate the LPS-induced inhibition of proliferation and apoptosis in IEC-6 cells.

### 2.5. Cow Colostrum and Mature Milk EVs Reduced LPS-Induced Proinflammatory Cytokines-Related Gene Expression in IEC-6 Cells

**Figure 7:**
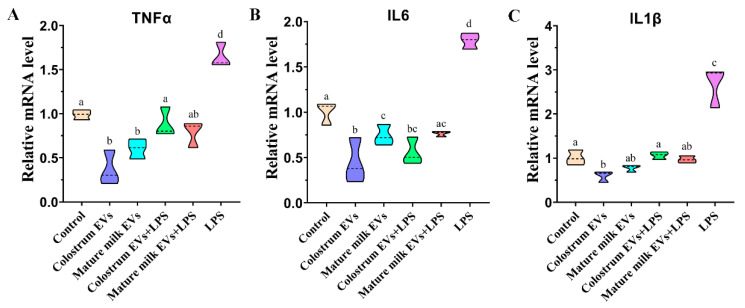
Effects of cow colostrum and mature milk EVs supplementation on inflammatory-related genes expression in IEC-6 cells. The mRNA levels of *TNFα* (**A**), *IL6* (**B**), and *IL1β* (**C**) were examined by real-time PCR in IEC-6 cells at 24 h after EVs supplementation. After 24 h of incubation, cells were treated with 0.5 μg/mL LPS for 12 h. The quantity of mRNA was normalized to that of β-actin. The statistical differences were performed using one-way ANOVA. Bars with different lowercase letters indicate significant differences (*p* < 0.05). The experiment was repeated three times independently.

To further verify the effects of colostrum and mature milk EVs on proinflammatory cytokines-related gene expression, we tested the expression of *TNFα*, *IL6,* and *IL1β* after EVs and LPS treatment. As Figure 7 shows, compared with control, LPS significantly increased the expression of *TNFα*, *IL6,* and *IL1β* in IEC-6 cells and colostrum and mature milk EVs significantly decreased the expression of *TNFα*, *IL6,* and *IL1β* (besides the mature milk EVs on *IL1β*, *p* < 0.05, Supplementary Table S6). The change in *TNFα* and *IL1β* was not different between the colostrum EVs group and the mature milk EVs group (*p* > 0.05). In addition, colostrum and mature milk EVs significantly attenuated the effect on increasing the expression of *TNFα*, *IL6,* and IL1β induced by LPS (*p* < 0.05). These results suggested that colostrum and mature milk EVs could attenuate the IEC-6 cells’ proinflammatory responses induced by LPS.

### 2.6. Colostrum and Mature Milk EVs Relieve LPS-Induced Dysregulation of the Expression of Barrier-Related Genes in IEC-6 Cells

**Figure 8:**
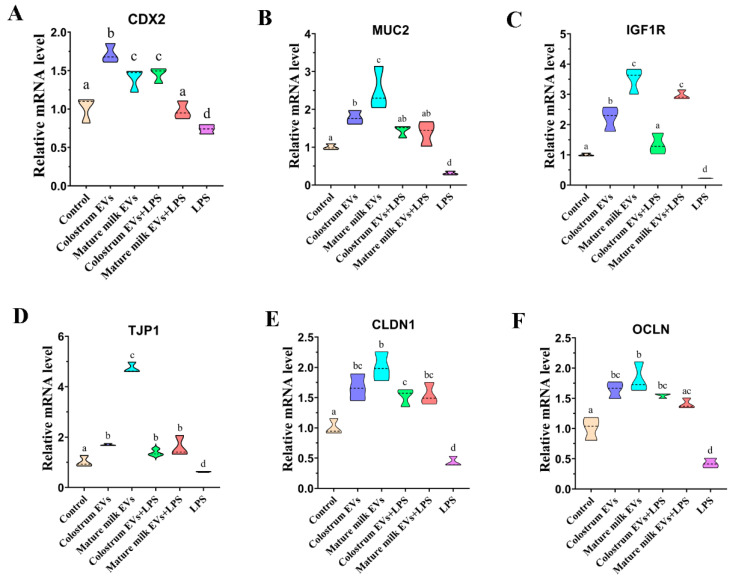
Effects of cow colostrum and mature milk EVs supplementation on the expression of barrier-related genes in IEC-6 cells. The mRNA levels of *CDX2* (**A**), *MUC2* (**B**), *IGF1R* (**C**), *TJP1* (**D**), *CLDN1* (**E**), and *OCLN* (**F**) were examined by real-time PCR in IEC-6 cells at 24 h after EVs supplementation. After 24 h of incubation, cells were treated with 0.5 μg/mL LPS for 12 h. The quantity of mRNA was normalized to that of β-actin. The statistical differences were performed using one-way ANOVA. Bars with different lowercase letters indicate significant difference (*p* < 0.05). The experiment was repeated three times independently.

The intestinal barrier is essential to the intestine functions. Therefore, we investigated the expression of intestinal barrier-related genes in IEC-6 cells (Figure 8, Supplementary Table S6). The present results indicated that both colostrum EVs and mature milk EVs could increase the expression of *TJP1*, *CLDN1*, *OCLN*, *CDX2*, *MUC2,* and *IGF1R* compared with control (*p* < 0.05). Mature milk EVs significantly promoted the expression of *MUC2*, *TJP1,* and *IGF1R* compared to that in the colostrum EVs group (*p* < 0.05), and colostrum EVs significantly increased the expression of *CDX2* compared with the mature milk EVs group (*p* < 0.05). Moreover, LPS significantly decreased the expression of *TJP1*, *CLDN1*, *OCLN*, *CDX2*, *MUC2,* and *IGF1R* (*p* < 0.05). However, colostrum and mature milk EVs relieved the LPS-induced inhibition on the expression of *TJP1*, *CLDN1*, *OCLN*, *CDX2*, *MUC2,* and *IGF1R* (*p* < 0.05). These results suggested that colostrum and mature milk EVs had beneficial effects on protecting the intestine functions by strengthening the intestinal barrier.

### 2.7. Colostrum and Mature Milk EVs Changed the miRNAs Expression in IEC-6 Cells

**Figure 9:**
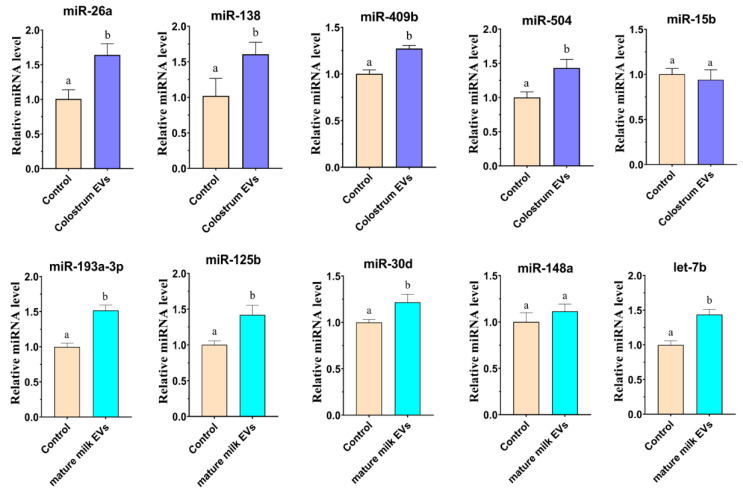
Effects of cow colostrum and mature milk EVs supplementation on the miRNAs expression in IEC-6 cells. The miRNAs levels (miR-26a, miR-138, miR-409b, miR-504, miR-15b, miR-193a-3p, miR-125b, miR-30d, miR-148a, and let-7b) were examined by real-time PCR in IEC-6 cells at 24 h after EVs supplementation derived from colostrum and mature milk. The quantity of miRNAs was normalized to that of U6. Bars with different lowercase letters indicate significant difference (*p* < 0.05). The experiment was repeated three times independently.

To testify that milk EVs miRNA could enter into IEC-6 cells and change its miRNAs expression, the expression of miR-26a, miR-138, miR-409b, miR-504, and miR-15b was determined in IEC-6 cells after treatment with colostrum EVs, and the level of miR-193a-3p, miR-125b, miR-30d, miR-148a, and let-7b was tested in IEC-6 cells after treatment with mature milk EVs (Figure 9, Supplementary Table S6), respectively. The results showed that the level of miR-26a, miR-138, miR-409b, miR-504, and miR-15b was significantly higher in IEC-6 cells after treatment with colostrum EVs than with control (*p* < 0.05). Similarly, mature milk EVs significantly increased the level of miR-193a-3p, miR-125b, miR-30d, and let-7b in IEC-6 cells compared with control (*p* < 0.05). These results indicated that EVs derived from colostrum and mature milk could be taken up by IEC-6 cells and then increase miRNAs expression in IEC-6 cells.

## 3. Discussion

Multiple studies have indicated that milk EVs play an important role in protecting the intestine, including attenuation of LPS-induced inflammation, inhibiting apoptosis, promoting cell proliferation, and inducing tight junction proteins formation, and effectively activate the hypoxia-inducible factor signaling pathway [2,5,39,40,41]. Thus, it will be of great importance to examine the difference between cow colostrum and mature milk EVs; how colostrum and mature milk EVs alter IEC-6 cells’ function and composition of functional miRNAs; and how those modifications of IEC-6 cells and colostrum and mature milk EVs regulate inflammation of IEC-6 cells induced by LPS. In the present study, EVs were isolated from cow colostrum and mature milk. The differences between colostrum and mature milk EVs, including the number of EVs, EVs protein concentration, miRNAs, proliferation, apoptosis, proinflammatory cytokines, and intestinal barrier-related genes induced by LPS in IEC-6 cells, were compared after EVs added to IEC-6 cells. The results showed that milk EVs could attenuate the damage on IEC-6 induced by LPS. Moreover, colostrum and mature milk EVs exhibited differential characteristics that affected the IEC-6 cells’ function.

Intestinal inflammatory disease is common because intestinal epithelial cells are normally exposed to intact bacteria and its products. However, the intestinal epithelium plays a crucial role in forming an essential barrier and provides the host defense against pathogens [8,42]. In addition, intestinal epithelial cells can quickly respond to intestinal invasion as the first component of the innate immune system [7]. The imbalance between proinflammatory and anti-inflammatory effect in intestinal epithelial cells is a major cause of the loss of the epithelial barrier [43]. However, how to maintain health and reduce inflammation in the gastrointestinal tract is still a matter of concern. Accumulated evidence has shown that EVs play an important role in regulating the recipient cells’ function. The main reason is that EVs contain large amounts of mRNAs, miRNAs, noncoding RNAs, as well as proteins [44]. Moreover, milk EVs can be taken up by the intestinal cells via endocytosis, pass through the intestinal mucosa, and then play a role in biologically activating the response signal [2,45,46,47]. Interestingly, porcine milk EVs protect the intestine epithelial cells against LPS-induced injury by inhibiting cell inflammation and protecting against apoptosis through TLR4/NF-κB and p53 pathway and facilitate the development of the mouse intestine and the proliferation of intestinal epithelial cells [5,41]. Further, yak-milk-derived EVs significantly inhibit p53 levels, thus promoting IEC-6 cell survival [2,39]. Similarly, research indicates that milk EVs may cross intestinal epithelial cells and reach the systemic circulation, where milk EVs are able to facilitate intestinal cell proliferation and promote tract development [2,41,48]. Human breast milk-derived EVs also could attenuate cell death in intestinal epithelial cells [49]. Dairy cow colostrum-derived EVs activate the proliferation of colonic epithelial cells and create an environment to relieve inflammation by effectively removing reactive oxygen species and regulating the expression of immune cytokines [50]. In line with previous studies, our results suggested that cow colostrum and mature milk EVs could promote IEC-6 cells proliferation and inhibit the IEC-6 cells apoptosis, and attenuate LPS-induced inhibition of proliferation and promotion of apoptosis in IEC-6 cells. It has been proved that local inflammation could seriously destroy intestine cell permeability [51]. Stremmel et al. [52] provided experimental evidence of the pronounced anti-inflammatory effects of bovine milk EVs in a murine genetic colitis model. Human milk-derived EVs induce proliferation- and epithelial mesenchymal transformation-related changes in normal colonic epithelial cells but not in colonic tumor cells [53]. Moreover, cow and human milk-derived EVs have a therapeutic and anti-inflammatory effect on colitis induced by dextran sulfate sodium in murine Model [54]. Similarly, oral administration of EVs derived from milk effectively suppress excessive gut inflammatory responses and improve gut barrier integrity in dextran sulfate sodium-induced acute and chronic gut inflammation and diet-induced experimental NASH [55]. Tong et al. [56] also provided evidence that abundant proteins and microRNAs in EVs from milk were involved in the regulation of immune and inflammatory pathways and that oral administration of EVs from milk prevented colon shortening, reduce intestinal epithelium disruption, and inhibited infiltration of inflammatory cells and tissue fibrosis in a mouse ulcerative colitis model. EVs derived from bovine and human breast milk exert protective effects on epithelial tight junction functionality in vitro, survive harsh gastrointestinal conditions ex vivo, and reach the colon in vivo [55,57]. The present results also showed that milk EVs could decrease the inflammatory factors expression in IEC-6 cells induced by LPS and exhibited a beneficial anti-inflammatory effect. Compared with mature milk EVs, colostrum-derived EVs had a more efficient effect on suppressing the expression of *Caspase-3*, *p53,* and *IL6*. This indicates that milk EVs were able to protect IEC-6 cells from apoptosis and inflammation and colostrum-derived EVs exhibited more efficient effects. As for the intestinal cell, proliferation is the primary driver for growth and development [58]. Furthermore, balance between proliferation and apoptosis is important for epithelial cell renewal [59,60]. Therefore, colostrum and mature milk EVs could exert beneficial effects on protecting intestinal health by promoting proliferation and inhibiting apoptosis and inflammation induced by LPS. 

The epithelial barrier integrity is important for epithelial function. Once the epithelial barrier is damaged, intestinal bacteria can break through the barrier, enter the blood and cause an inflammatory response [6]. Maintaining the normal barrier function of epithelial cells is related to the balance among proliferation, apoptosis, inflammatory response, injury, and cytotoxicity [61]. In the tight junction protein (TJP1, CLDN1 and OCLN), Cdx2, mucus, and IGF1R are responsible for the construction of intestinal barrier structures and protect the intestinal epithelial cells from injury [40,62,63,64]. It has been demonstrated that bovine milk EVs exert their beneficial effects on necrotizing enterocolitis prevention in experimental mice by improving goblet cell expression and MUC2 (mucin2) production [65]. MUC2 is secreted into the gut lumen and is responsible for construction of the mucus barrier to protect the intestinal tract [62,66]. Cdx2 (caudal-related homeobox transcription factor2), an intestinal-specific transcription factor, is strongly required for maintenance of intestinal identity and is essential to the development of the murine intestinal tract, where it also regulates MUC2 expression involved in intestinal cell proliferation and differentiation [67,68,69]. Porcine milk EVs significantly enhance the tight junction genes expression (*TJP1*, *CLDN1,* and *OCLN*) and could significantly rescue tight junction genes expression inhibited by deoxynivalenol [40]. It is suggested that human milk EVs could contribute to the development of intestinal barrier function by inducing *TJP1* expression in the infant intestine and can protect infants from diseases [70]. Consistent with these studies, *TJP1*, *CLDN1*, *OCLN*, *Cdx2*, *MUC2,* and *IGF-1R* expression were significantly inhibited during continuous LPS exposures but could be significantly rescued by colostrum and mature milk EVs. This indicates that colostrum and mature milk EVs were able to effectively prevent the damage to the intestinal epithelial barrier function induced by LPS. Hence, the present study proposed that the beneficial intestinal epithelial barrier protection effect of EVs is related to mediating the expression of *TJP1*, *CLDN1*, *OCLN*, *Cdx2*, *MUC2,* and *IGF-1R*. Cow colostrum and mature milk play essential roles in regulating the immune function and development in calves and humans, due to the benefits of various nutritional and immunological components [22,71,72]. In addition, the enriched miRNAs in milk EVs also modify the recipient cells’ signaling and function [2,5,39,41]. Moreover, the enriched miRNAs in the milk EVs consist of rich amounts of immune-related miRNAs and play important roles in immune cell development [37,55,56,73,74]. In the present study, compared with mature milk EVs, we found 185 miRNAs were significantly downregulated and 144 miRNAs were significantly upregulated in the EVs derived from colostrum. Among them, bta-miR-106a, bta-miR-130a, bta-miR-130b, bta-miR-135b, bta-miR-149-5p, bta-miR-150, bta-miR-15b, bta-miR-181c, bta-miR-181d, bta-miR-195, bta-miR-21-3p, bta-miR-223, bta-miR-26a, bta-miR-30b-3p in colostrum EVs and bta-let-7a-3p, bta-let-7b, bta-miR-148a, bta-miR-15a, bta-miR-186, bta-miR-22-3p, bta-miR-29b, and bta-miR-29c in mature milk EVs were associated with the immune system [18,37,75,76]. Importantly, colostrum EVs could significantly promote the level of bta-miR-26a, bta-miR-138, bta-miR-409b, bta-miR-504, and bta-miR-15b in IEC-6 cells after treatment with colostrum EVs. Similarly, mature milk EVs also significantly induced the expression of bta-miR-193a-3p, bta-miR-125b, bta-miR-30d, and bta-let-7b in IEC-6 cells after milk EVs supplement. Furthermore, the differentially expressed miRNAs were involved in many important signal pathways, such as cell growth and death, signal transduction, signaling molecules and interaction, infectious disease, immune system, development and regeneration and so on. Moreover, we found that 36 miRNAs, ranking among the top 40 most abundant miRNAs involved in signaling pathways related to intestinal barrier integrity, were present in both cow colostrum and mature milk EVs. Verma [77] identified 154 differentially expressed miRNAs in human colostrum and milk EVs, whereas 49 miRNAs were revealed as immune-related miRNAs. These miRNAs were also abundantly present in dairy cow colostrum and mature milk EVs in our study. Importantly, these miRNAs are remarkable, with an evolutionary conserved character of a selected set of miRNAs in milk EVs. This suggests that they play a possible conserved role in regulating the newborn’s epithelial barrier and immune system, contributing to the guided further development of the newborn [31]. Therefore, the enriched miRNAs in colostrum and milk EVs may be associated with the role of EVs in mediating the function of proliferation, apoptosis, inflammatory cytokines, and barrier protection in IEC-6 cells. Besides the differentially expressed miRNAs, we also explored the difference between colostrum and mature milk EVs in regulating proliferation, apoptosis, proinflammatory cytokines, and the epithelial barrier in IEC-6 cells. The results indicated that EVs derived from colostrum had better effects on inhibiting the apoptosis and proinflammatory cytokines-related genes expression. However, the EVs derived from mature milk exhibited beneficial effects on intestinal epithelial barrier protection.

## 4. Materials and Methods

### 4.1. Milk Samples

We collected 5 colostrum (days 3 postpartum) and 5 mature milk (beyond day 30 postpartum) samples from the lactating Holstein dairy with 1 parity in a commercial dairy farm in the Hebei province of China. The feeding and management of cows were performed as previously described [78]. Simply, the cows were provided with total mixed ration at 08:00, 16:00, and 24:00. Moreover, the cows were milked in a milk carousel with 80 milk stalls at 07:00, 15:00, and 23:00, respectively. About 40 mL of milk was sampled. The samples were immediately sent back to the laboratory in dry ice and kept at −80 °C until use.

### 4.2. EVs Preparation

Five colostrum EVs and five mature milk EVs were isolated by serial centrifugations at 4 °C according to previous literature [2,23,24,79]. Briefly, the milk was centrifuged (5000× *g*, 30 min, 4 °C) to aspirate the fat layer, large debris and cells. The defatted supernatant was transferred to a new tube and centrifuged again (12,000× *g*, 30 min, 4 °C) to eliminate residual fat, as well as somatic and cell debris. An equal volume of 0.25 M EDTA (pH 7) was added to the new supernatant and incubated on ice for 15 min to precipitate casein and EVs coated with casein, as described by Kusuma et al. [45]. The clear supernatant was then filtered by 0.45 μm and 0.22 μm filters and ultracentrifuged (120,000× *g*, 90 min, 4 °C, Beckman, SW41T rotor, CA, USA). The pelleted EVs were resuspended in PBS and then ultracentrifuged (120,000× *g*, 90 min, 4 °C) for washing. Finally, the EVs were resuspended in PBS and then stored at −80 °C until use.

### 4.3. Transmission Electron Microscopy

The samples were deposited into formvar-coated copper grids for 2 min. Subsequently, the samples were negatively stained with 2% uranyl acetate for 1 min. Then, the grids were washed by transferring the samples onto drops of double-distilled water and the excess liquid was removed by a filter paper. Finally, the EVs were observed by a transmission electron microscope at 100 kV (H-7650, HITACHI, Tokyo, Japan).

### 4.4. EVs Protein Quantification

The colostrum and mature milk EVs protein concentration was measured with the BCA Protein Assay Kit using bovine serum albumin (BSA) as standard according to the manufacturer’s instructions (Beyotime Institute of Biotechnology, Shanghai, China). All 10 EVs samples collected from cow’s milk were used to detect the protein concentration and were tested 3 times.

### 4.5. EVs Characterization and Quantification

Five colostrum EVs and five mature milk EVs’ concentrations and size distribution were analyzed using the nFCM (Nano-flow cytometry, Xiamen, China) according to the reported protocols [79,80,81]. Briefly, two single-photon counting avalanche photodiodes (APDs) were used for the simultaneous detection of side scatter (SSC) and fluorescence of individual particles. The instrument was calibrated for particle concentration using 200 nm PE and AF488 fluorophore conjugated polystyrene beads and for size distribution using Silica Nanosphere Cocktail containing nanosphere populations of 68 nm, 91 nm, 113 nm, and 155 nm in diameter (NanoFCM Inc., S16M-Exo, Xiamen, China). Any particles that passed by the detector during a 1 min interval were recorded in each test. All samples were diluted to obtain a particle count within the optimal range of 2000–12,000/min and samples injection pressure was maintained at 0.8 kPa, yielding well-separated particle detection events. Using the calibration curve, the flow rate and side scattering intensity were converted into corresponding vesicle concentration and size on the NanoFCM software (NanoFCM Profession V1.0).

### 4.6. Small RNA Sequencing

Total RNAs were isolated from 3 colostrum EVs and 3 mature milk EVs using QIAzol Lysis Reagent (QIAGEN, Valencia, CA, USA) for miRNA sequencing. Small RNA regions with the 18–30 nt were gel purified using a 15% urea PAGE. Subsequently, these purified small RNAs were ligated to 3′ Adapter and 5′ Adapter. Then, the adapter-ligated products were reverse transcribed using SuperScript II Reverse Transcriptase (Invitrogen, CA, USA) and amplified by 15 cycles rounds of PCR to enrich the cDNA fragments. Next, 100–120 bp fragments were selected from the gel and purified using the QIAquick Gel Extraction Kit (QIAGEN, Valencia, CA, USA). The distribution of the fragment sizes obtained was checked by an Agilent 2100 bioanalyzer (Agilent, MA, USA). The purified cDNA library was denatured, which provided the final miRNA library. Subsequently, miRNA profiling was sequenced on the BGISEQ-500 platform (BGI, Wuhan, China). After filtering, the raw sequencing data were mapped to the Bos taurus reference genome (ARS-UCD1.2) and other sRNA databases including miRbase, siRNA, piRNA, and snoRNA with Bowtie2 [82]. Differential expression analysis was calculated by the DESeq on Dr. Tom Multi-omics Data Mining System (https://biosys.bgi.com, 6 May 2023) according to the criteria of Q value ≤ 0.05 and |log2FC| > 1 [83]. RNAhybrid [84], miRanda [85] and TargetScan [86] were applied to predict target genes of miRNAs. For annotation, all target genes were aligned against the Kyoto Encyclopedia of Genes KEGG and Gene Ontology database and performed using phyper, a function of R on Dr. Tom Multi-omics Data Mining System (https://biosys.bgi.com, 6 May 2023). A *p*-value ≤ 0.05 was used to define significantly enriched terms.

### 4.7. IEC-6 Cell Culture

The IEC-6 cells were obtained from Hunan Fenghui Biotechnology Co., Ltd (Hunan, China). The cells were cultured in Dulbecco’s Modified Eagle Medium (DMEM) (Gibco, Grand Island, NY, USA) containing 10% fetal bovine serum (FBS; Hyclone, UT, USA) and 50 µg/mL of streptomycin and penicillin (Pen-Strep, Invitrogen, Carlsbad, CA, USA). The IEC-6 cells were finally cultured in 12-well plates in an incubator at 37 °C; the incubator contained 5% CO_2_. For cell treatment, the IEC-6 cells were assigned to the DMEM group (Control), colostrum EVs group, mature milk EVs group, the colostrum EVs + LPS group, the mature milk EVs + LPS group, and the LPS group. When the seeded IEC-6 cells reached 70–80% confluency, the cells were treated with 100 µg/mL of colostrum and mature milk EVs. After 24 h of incubation, the LPS group and EVs + LPS group were treated with 0.5 μg/mL LPS for 12 h. The IEC-6 cells were collected for further research. In this study, the experimental protocols were reviewed and approved by the Huazhong agriculture university Institutional Committee on Animal Care and Use.

### 4.8. Detection of Expression of miRNAs by Real-Time PCR

The total miRNA from EVs or IEC6 cells was extracted using miRcute miRNA Isolation Kit (Tiangen, Beijing, China) according to the manufacturer’s instructions and then the total RNA was reverse transcribed using a miRcute Plus miRNA First-Strand cDNA Kit (Tiangen, Beijing, China). The mature miRNAs were quantified by quantitative real-time PCR using the miRcute Plus miRNA qPCR Kit (SYBR Green). Briefly, quantitative real-time PCR was performed on a LightCycler 480Ⅱ Real-Time PCR System (Roche, Penzberg, Germany) containing 2 × miRcute Plus miRNA PreMix (5 μL), specific primer (0.2 μM for each primer), reverse-transcribed cDNA (1 μL), and RNase and DNase-free water ddH_2_O (3.6 μL). Amplification was performed as follows: 95 °C for 15 min, 40 cycles at 94 °C for 20 s, 60 °C for 34 s; and a melting curve analysis was performed from 65 °C to 95 °C to confirm specific PCR products. The 5′-specific primers used for detecting miRNAs are listed in Table S1. Finally, the relative expression of miRNA was normalized against U6 levels and the expression levels were analyzed using the 2^−∆∆CT^ method [87].

### 4.9. Detection of Expression of mRNA by Real-Time PCR

IEC-6 cells were harvested after treatment with colostrum and mature milk EVs and LPS. The total RNA was extracted using AG RNAex pro Reagent (Accurate Biotechnology Co., Ltd, Changsha, China) and reverse transcribed with oligo (dT) using a cDNA Synthesis Kit (Thermo Scientific, Waltham, MA, USA). Briefly, quantitative real-time PCR was performed on a LightCycler 480Ⅱ Real-Time PCR System (Roche, Penzberg, Germany) in a final 10 μL volume reaction, containing 5 μL of LightCycler 480 SYBR Green Ⅰ Master Mix, 0.5 μM of specific primer, 1 μL of cDNA and 3 μL of RNase and DNase-free water ddH_2_O. Amplification was performed as follows: 5 min at 95 °C, 40 cycles of 20 s at 95 °C, 20 s at corresponding annealing temperatures and 72 °C for 20 s, and a melting curve analysis was performed from 65 °C to 95 °C to confirm specific PCR products. The primers designed for detecting mRNA are listed in Table S1. Finally, the relative expression of mRNAs was normalized to β-actin levels, and the expression levels were analyzed using the 2^−∆∆CT^ method [87].

### 4.10. Western Blot Analysis

The EVs and IEC-6 cells were lysed using RIPA containing protease and phosphatase inhibitor cocktail (Thermo Scientific, Rockford, IL, USA). The total protein was loaded onto gels and separated by 12% polyacrylamide gel electrophoresis, and then blotted onto polyvinylidene fluoride membrane (Millipore, Bedford, MA, USA). Firstly, the membranes were blocked and incubated overnight with primary mouse monoclonnal antibodies against CD63 (sc-5275, Santa Cruz, Dallas, TX, USA), CD81 (sc-23962, Santa Cruz, Dallas, TX, USA), Bcl2 (sc-7382, Santa Cruz, Dallas, TX, USA), Bax (sc-7480, Santa Cruz, Dallas, TX, USA), Caspase-3 (sc-56053, Santa Cruz, Dallas, TX, USA), p53 (sc-126, Santa Cruz, Dallas, TX, USA), β-actin (sc-47778, Santa Cruz, Dallas, TX, USA) at 4 °C. Subsequently, the blots were incubated with HRP-labeled anti-mouse secondary antibody (SC-2005, 1:5000; Santa Cruz, Dallas, TX, USA). Finally, the bands were visualized using Clarity Western ECL kit according to the manufacturer’s instructions (Bio-Rad Laboratories, Hercules, CA, USA) and exposed using a ChemiDocXRS chemiluminescent imaging system (Bio-Rad, Hercules, CA, USA).

### 4.11. Cell Proliferation Tested by EdU Assay

IEC-6 cells were seeded in 24-well plates at a density of 2.5 × 10^4^/cm^2^. At the end of the treatment with EVs and LPS, the IEC-6 cells used for EdU were labeled by BeyoClick™ EdU Cell Proliferation Kit with Alexa Fluor 488 (Beyotime, Shanghai, China) according to the manufacturer’s protocol. The following steps were carried out according to the manual of the BeyoClick™ EdU Cell Proliferation Kit. After treatment, 20 μM of the EdU labeling medium was added to the cell culture for incubation for 2 h at 37 °C with 5% CO_2_. After EdU labeling, cultured IEC-6 cells were fixed with 4% paraformaldehyde for 15 min and then the cells were washed with 3% BSA in PBS 3 times. IEC-6 cells were permeabilized with 0.5% TritonX-100 in PBS for 15 min and then the cells were washed with 3% BSA in PBS 3 times. Finally, the cells were incubated with Hoechst 33342 dye at room temperature for 10 min and washed 3 times. Then, the EdU-positive IEC-6 cells were observed using a Nikon TE2000-U inverted microscope (Nikon Instruments, Tokyo, Japan) and counted by Image Pro Plus (Media Cybernetics, Inc., Silver Spring, MD, USA).

### 4.12. Cell Apoptosis Assay

The apoptosis rate of IEC-6 cells was detected by flow cytometry. After treatment, the IEC-6 cells were harvested by trypsinization and washed with PBS. The IEC-6 cells were labeled with 5 μL Annexin V-FITC and 10 μL propidium iodide (PI) (Beyotime Biotechnology, Shanghai, China) for 15 min at room temperature in darkness and analyzed by flow cytometry (ACEA Biosciences, CA, USA). The cells apoptosis was analyzed by NovoExpresst software 1.3 (ACEA Biosciences). The experiments were performed in triplicate.

### 4.13. Statistical Analysis

The data were presented as the mean ± standard deviations (SD) of three replicates. Significance differences were determined using one-way ANOVA with SPSS 17, and treatment means were compared using Tukey’s test for post hoc multiple comparisons via SPSS 17 software. The *t*-test was used to determine the significance of differences between two groups. *p* < 0.05 was considered to represent a significant difference.

## 5. Conclusions

The present study identified differentially expressed miRNAs in EVs derived from cow colostrum and mature milk. These differentially expressed miRNAs contain rich amounts of immune-related miRNAs. In addition, they are also involved in many important signal pathways. The supplied EVs could alter the level of miRNAs in IEC-6 cells. This suggests that EVs could play an important role in regulating the recipient cells’ function by transferring their miRNAs. Moreover, colostrum and mature milk EVs both inhibited the level of apoptosis and proinflammatory cytokines and promoted the proliferation and expression of barrier-related genes in IEC-6 cells. Only colostrum EVs inhibited the level of apoptosis-related genes. Importantly, EVs could attenuate the promotion of apoptosis and inflammatory factors expression induced by LPS in IEC-6 cells and reduce the inhibition of proliferation and intestinal epithelial barrier-related genes in IEC-6 induced by LPS. Furthermore, colostrum and mature milk EVs exhibited different characteristics in mediating the function of IEC-6 cells. These findings further highlight that EVs derived from colostrum and mature milk could affect the gut health and help us to understand the effect of milk EVs on the regulation of gastrointestinal tract development under LPS conditions.

## Data Availability

Data are contained within the article and Supplementary Materials.

## Supplementary Materials

The following supporting information can be downloaded at: https://www.mdpi.com/article/10.3390/ijms25073880/s1.
