# Supplementary material for: The Difference of Milk-Derived Extracellular Vesicles from Cow Colostrum and Mature Milk on miRNAs Expression and Protecting Intestinal Epithelial Cells against Lipopolysaccharide Damage
Source: Int J Mol Sci. 2024 Mar 30;25(7):3880. doi: 10.3390/ijms25073880 (PMC11011493; doi:10.3390/ijms25073880)
Supplement: Supplementary file 1 [file ijms-25-03880-s001.zip › ijms-2762790-supplementary.pdf]

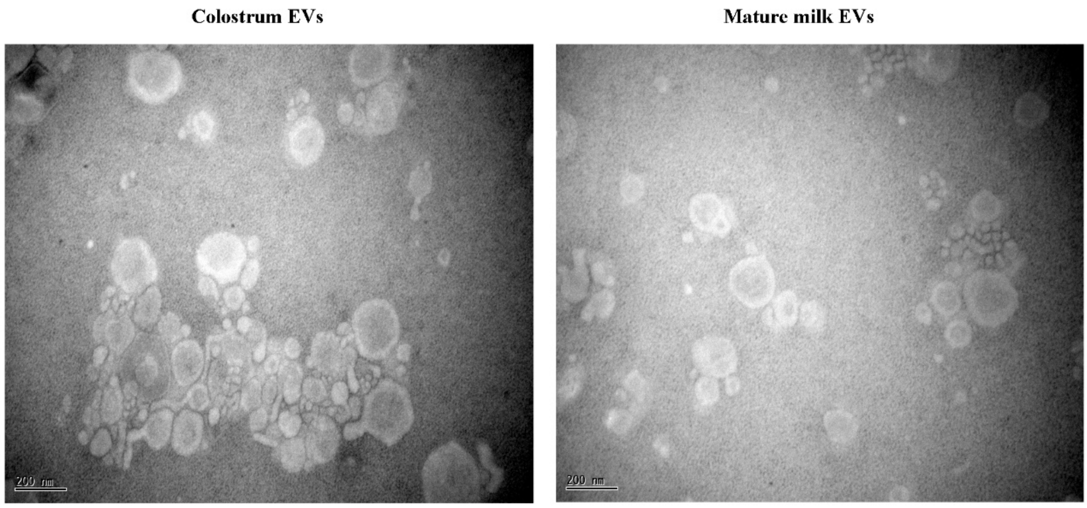

Figure S1. The TEM of colostrum and mature milk-derived EVs.

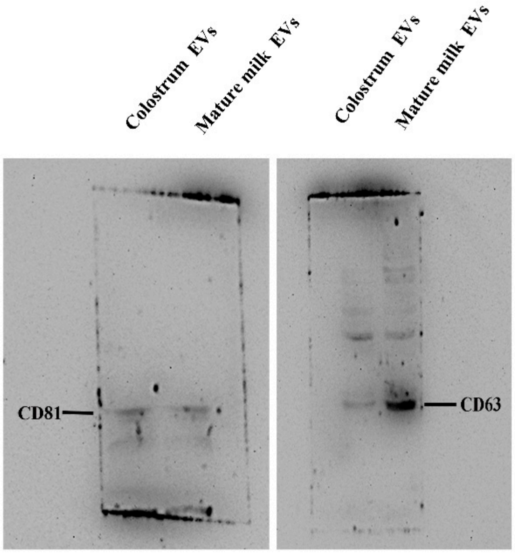

Figure S2. The colostrum and mature milk-derived EVs protein markers (CD81 and CD63) detected by Western blot.

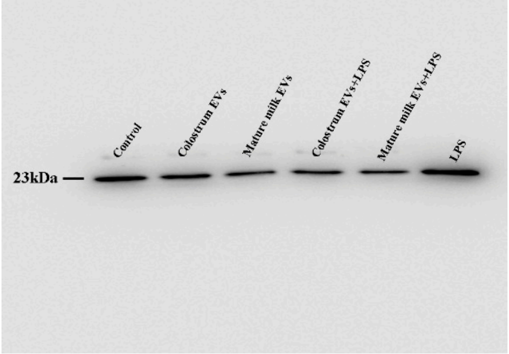

Figure S3. The Bax protein level in IEC-6 cells after colostrum EVs, mature milk EVs and LPS treatment detected by Western blot.

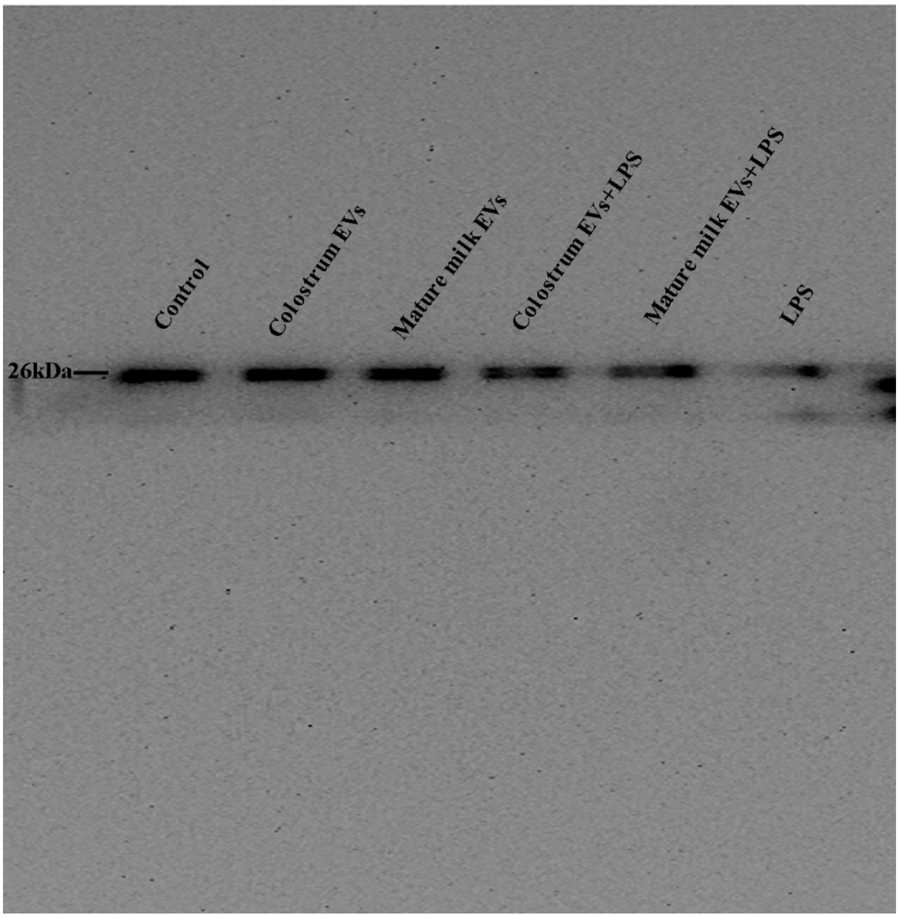

Figure S4. The Bcl2 protein level in IEC-6 cells after colostrum EVs, mature milk EVs and LPS treatment detected by Western blot.

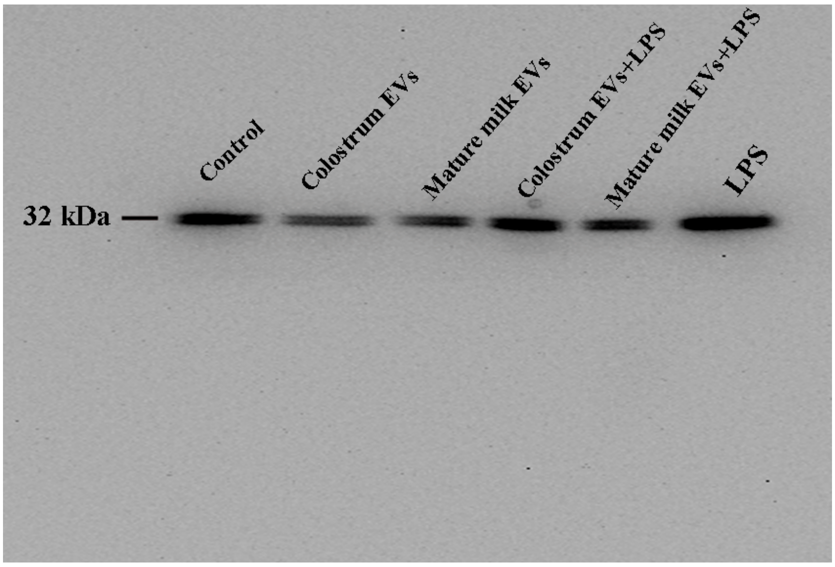

Figure S5. The Caspase-3 protein level in IEC-6 cells after colostrum EVs, mature milk EVs and LPS treatment detected by Western blot.

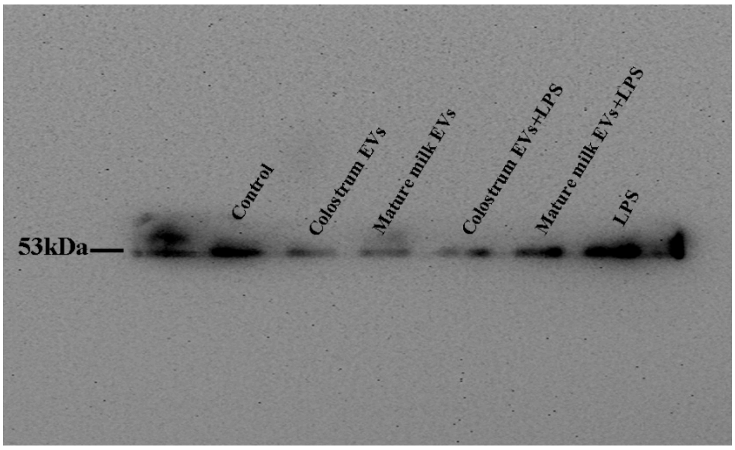

Figure S6. The P53 protein level in IEC-6 cells after colostrum EVs, mature milk EVs and LPS treatment detected by Western blot.

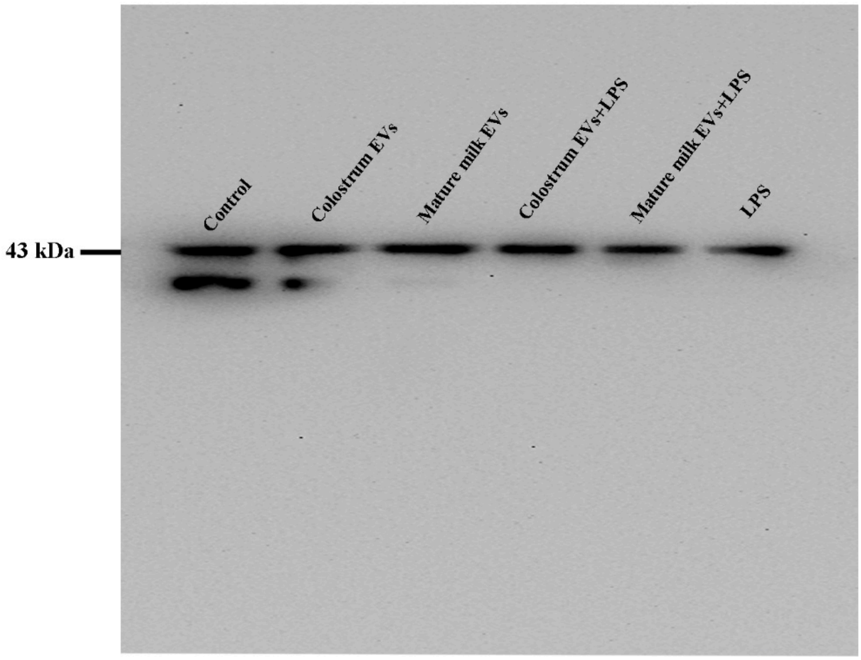

Figure S7. The β-actin protein level in IEC-6 cells after colostrum EVs, mature milk EVs and LPS treatment detected by Western blot.
