# Supplementary material for: The Difference of Milk-Derived Extracellular Vesicles from Cow Colostrum and Mature Milk on miRNAs Expression and Protecting Intestinal Epithelial Cells against Lipopolysaccharide Damage
Source: Int J Mol Sci. 2024 Mar 30;25(7):3880. doi: 10.3390/ijms25073880 (PMC11011493; doi:10.3390/ijms25073880)
Supplement: Supplementary file 1 [file ijms-25-03880-s001.zip › Table S1.pdf]

Table S1. Sequences of primer pairs for quantitative real-time PCR.

Primer	Forward	Reverse
Bax	AGGATGCGTCCACCAAGAA	CAAAGTAGAAGAGGGCAACCAC
Caspase-3	TGACTGGAAAGCCGAAACTC	GCAAGCCATCTCCTCATCAG
P53	CGACCTATCCTTACCATCATC	GGGTGAAATACTCTCCATCAAG
Bcl2	GGCATCTGCACACCTGGAT	ATCAAACAGAGGTCGCATGCT
TNF-α	ACG GCA TGG ATC TCA AAG AC	GTG GGT GAG GAG CAC GTA GT
IL-6	CTG CAA GAG ACT TCC ATC CAG TT	GAA GTA GGG AAG GCC GTG G
IL-1β	CAG GCA GGC AGT ATC ACT CA	TGT CCT CAT CCT GGA AGG TC
Cdx2	AGCCAAGTGAAAACCAGGAC	AGTGAAACTCCTTCTCCAGCTC
MUC2	TTT CAA GCA CCC CTG TAA CC	AGG TCC TGG TGT TGA ACC TG
TJP1	ACT ATG ACC ATC GCC TAC GG	GGG GAT GCT GAT TCT CAA AA
CLDN1	AGATACAGTGCAAAGTCTTCGA	CAGGATGCCAATTACCATCAAG
OCLN	CAGGATGCCAATTACCATCAAG	GGGTTCACTCCCATTATGTACA
IGF1R	GGCAAGTATGCGTGAAAGAATC	CTAAAGGTCGGAGGAATGAGG
β-actin	TGCTGTCCCTGTATGCCTCT	CTTTGATGTCACGCACGATTT
**bta-miR-362-3p**	CGCCGGAACACACCTATTCA	
**bta-miR-122**	ACCACCGTGGAGTGTGACAA	
**bta-miR-23b-3p**	AACCGGATCACATTGCCAGG	
**bta-miR-202**	CGCGGCCTTCCTATGCATAT	
**bta-miR-760-5p**	AACAATCCCCTCAGTCCACCA	
**bta-miR-32**	ACGCCGTATTGCACATGACT	
**bta-miR-21-3p**	AACCGGAACAGCAGTCGATG	
**bta-miR-503-5p**	AACAAGTAGCAGCGGGAACAG	
**bta-miR-377**	ACCACCGATCACACAAAGGC	
**bta-miR-211**	ACGCCGTTCCCTTTGTCATC	
**bta-miR-26a**	ACGCCGTTCAAGTAATCCAGG	
**bta-miR-138**	AACACGCAGCTGGTGTTGTG	
**bta-miR-409b**	AACAATGGGGTTCACCGAGC	
**bta-miR-504**	AACAAGAGACCCTGGTCTGCA	
**bta-miR-193a-3p**	AACCGGAACTGGCCTACAAAG	
**bta-miR-125b**	AACAAGTCCCTGAGACCCTAACTTG	
**bta-miR-30d**	AACAAGTGTAAACATCCCCGACTG	
**bta-miR-148a**	AACAAGTCAGTGCACTACAGAACTT	
**bta-let-7b**	AACACGCTGAGGTAGTAGGTTGT	
**bta-miR-15b**	AACACGCTAGCAGCACATCAT	
**U6**	GCTTCGGCAGCACATATACTAAAAT	
